# Easy-Prime: a machine learning–based prime editor design tool

**DOI:** 10.1186/s13059-021-02458-0

**Published:** 2021-08-19

**Authors:** Yichao Li, Jingjing Chen, Shengdar Q. Tsai, Yong Cheng

**Affiliations:** 1 Department of Hematology, St. Jude Children’s Research Hospital, Memphis, TN USA; 2 Integrated Biomedical Sciences Program, University of Tennessee Health Science Center, Memphis, TN USA; 3 Department of Computational Biology, St. Jude Children’s Research Hospital, Memphis, TN USA

**Keywords:** Prime editor, Machine learning, pegRNA design

## Abstract

Prime editing is a revolutionary genome-editing technology that can make a wide range of precise edits in DNA. However, designing highly efficient prime editors (PEs) remains challenging. We develop Easy-Prime, a machine learning–based program trained with multiple published data sources. Easy-Prime captures both known and novel features, such as RNA folding structure, and optimizes feature combinations to improve editing efficiency. We provide optimized PE design for installation of 89.5% of 152,351 GWAS variants. Easy-Prime is available both as a command line tool and an interactive PE design server at: http://easy-prime.cc/.

**Supplementary Information:**

The online version contains supplementary material available at 10.1186/s13059-021-02458-0.

## Background

Genome-editing technologies have revolutionized genetic studies ranging from those involving traditional interventions to precise manipulations of DNA sequences, offering both simplicity and robust outcomes [1]. Among different genome-editing technologies, the clustered regularly interspaced short palindromic repeats (CRISPR)–based systems [2–4] are the most widely used ones. Different CRISPR-based systems have their own strengths and weaknesses. Standard CRISPR-Cas9 approaches tend to introduce imprecise edits with indels varying in size from a single nucleotide to hundreds of nucleotides through nonhomologous end joining (NHEJ) [4]. In contrast, base editors can generate transition point mutations with high efficiency and accuracy without introducing double-strand breaks [5–7]. However, base editors are not suitable for generating other types of point mutations or for insertions and deletions.

Prime editing, a newly invented genome-editing technology, enables all types of base transitions and transversions to be accomplished, as well as customized insertions (up to 44 nucleotides) and deletions (up to 80 nucleotides) [8]. The key components of a prime editor (PE) (Additional file 1: Fig. S1) are a Cas9 nickase fused with reverse transcriptase and a prime editing guide RNA (pegRNA), which brings the PE machinery to targeted sites through a standard single-guide RNA (sgRNA) sequence. Upon binding, the Cas9 nickase nicks the target strand and the reverse transcriptase uses the primer binding site (PBS) of the pegRNA to initiate the reverse transcription. The genetic information encoded in the reverse-transcription template (RTT) can then be copied into the targeted site. There are four different prime editing systems: PE1, PE2, PE3, and PE3b. The only difference between PE1 and PE2 is the Moloney Murine Leukemia Virus (M-MLV) reverse transcriptase (RT) fused to the Cas9 nickase. In the PE3 system, a nick is introduced into the non-edited strand by a nick gRNA (ngRNA) to increase the editing efficiency. To further improve editing purity [8], a ngRNA spacer is designed to match the edited sequences in the PE3b system so that it only binds to the edited DNA sequences. Prime editing has been applied in human [9], mouse [10], and plants [11] with promising results. However, one major issue in the current PE system is the pegRNA/ngRNA design, which is much more complicated than the design process associated with other precise editing methods. Several programs (PrimeDesign [12], PegFinder [13], primeedit.nygenome [14], PnB Designer [15], PINE-CONE [16], DeepPE [17], and PE-Designer [18]) have been developed to simplify the search for pegRNAs and ngRNAs for prime editing by following general design guidance proposed in the original prime editing paper [8]. However, how to unbiasedly optimize the combination of all of these features and identifying the most suitable sequence from a list of candidates remains problematic.

We developed a machine learning–based framework, Easy-Prime, to systematically evaluate how position and sequence features affect PE2 and PE3 activity. We further integrated different PE-associated features and quantitatively predicted the editing efficiency using models trained from multiple published PE data. In addition to known PE–associated features, our framework also identifies and incorporates previously unappreciated RNA folding features that are strongly associated with PE efficiency. Using Easy-Prime, we further optimized the PE design targeting 136,365 variants associated with healthy traits or disorders and validated 7 blood traits associated variants experimentally.

## Results

### Quantitatively modeling PE-associated features

**Fig. 1:**
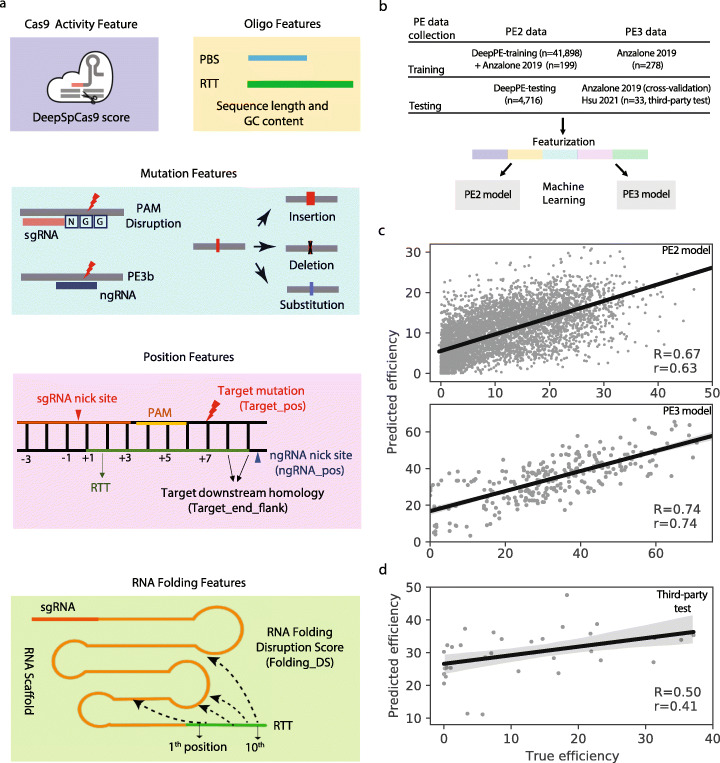
Overview of Easy-Prime design and machine learning model evaluation. **a** Cas9 activity feature is predicted by DeepSpCas9 score (purple box). (2) Oligo features (yellow box) are the GC content and sequence length of the PBS and RTT. (3) Target mutation features (cyan box) are whether the target mutation disrupts the PAM sequence, whether the ngRNA spacer sequence matches to the edited protospacer sequence, and the numbers of mismatches, deletions, and insertions. (4) Position features (pink box) are the distance between the ngRNA and the sgRNA (ngRNA_pos), the distance between the target mutation and the sgRNA (Target_pos), and the number of nucleotides downstream of the desired edit (target_end_flank). (5) RNA folding features are the maximal pairing probability between each of the first 10 bp of the RTT and the scaffold sequence based on RNAplfold [29]. **b** A machine learning workflow for data preprocessing, feature extraction, and model training and evaluation. **c** and **d** are correlation scatter plots of the true PE efficiency (*x*-axis) and the predicted efficiency (*y*-axis). **c** Train-test-split evaluation for the PE2 model and nested cross-validation evaluation for the PE3 model. **d** An independent PE data used for a third-party data evaluation for the PE3 model. “R” is spearman correlation coefficient. “r” is Pearson correlation coefficient

We selected 23 PE-related features in five categories (Fig. 1a, Additional file 2: Table S1): (1) the spCas9 activity feature predicted by DeepSpCas9 [19]); (2) oligo features, which include the length and GC content of the PBS and RTT; (3) target mutation features, which include mutation types such as single-nucleotide mutations or indels, and whether a target mutation disrupts the PAM sequence or the protospacer of the ngRNA (i.e., PE3b); (4) position features, which are the relative distances from the pegRNA nick site to the target mutation (Target_pos), from the pegRNA nick site to the ngRNA nick site (ngRNA_pos or nick position [8]), and from the target mutation to the end of the RTT (Target_end_flank or minimal homology downstream of the edit [12]); and (5) RNA folding features, which calculate the probability of different positions (i.e., the first 10 positions) on the RTT sequence disrupting the secondary structure of the RNA scaffold (i.e., the RNA-folding disruption score, see Methods).

We then trained machine learning models for PE2 and PE3 systems separately. For PE2 model, we used the 46,614 PE2 data generated by high-throughput integration system [17] and 199 endogenous editing sites measured by 597 amplicon sequencing [8]. For PE3 model, we used 278 endogenous editing sites measured by 829 amplicon sequencing [8] (Fig. 1b). We performed regression analysis using gradient boosting trees (GBTs, implemented using XGBoost [20]). We trained and evaluated the models by nested cross-validation, splitting the training set into training and validation sets based on target mutations (Additional file 1: Fig. S2). The editing efficiency predicted by our model correlated strongly with experimental measurements with 0.67 and 0.74 correlation coefficient (Spearman’s correlation) for the PE2 and PE3 systems, respectively (Fig. 1c). We further assessed the performance of our model using an independent dataset consisting of 33 PE3 data [12]. The correlation coefficient is 0.5 (Fig. 1d), suggesting a robust performance of Easy-Prime.

### Dissecting the features affecting PE efficiency

**Fig. 2:**
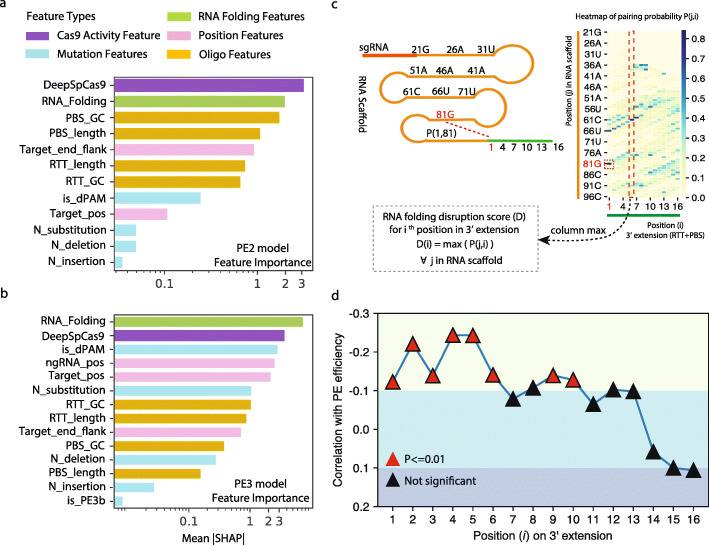
Features associated with PE efficiency. **a**, **b** Feature importance plot of the XGBoost regression model. Feature rankings are based on the mean absolute SHAP value for the PE2 and PE3 model. RNA folding features are combined for simplified visualization. Target_end_flank: number of nucleotides from target mutation to the end of RTT sequence. Target_pos: distance between target mutation and sgRNA nick site. ngRNA_pos: distance between ngRNA nick site and sgRNA nick site. **c** Schematic view of RNA-folding disruption score formulation. On the left, a pegRNA sequence consisting of an sgRNA (red), a scaffold sequence (orange), and an RTT sequence (green) is labeled with positions and nucleotides, such as 81G. The pairing probability between 81G and the first position in the RTT sequence is denoted as P(1,81). On the right is a heatmap of the pairing probability between each position in the scaffold and the 3′ extension sequence (i.e., RTT + PBS). P(1,81) is highlighted by a red dashed box. At bottom left, the formula to calculate D(i) is shown, where i represents the position in the 3′ extension. **d** Line plot showing the trend of correlations between the first 16 positions in the 3′ extension and the targeted editing frequency

Next, we quantitatively assessed the contribution of each feature using SHAP [21], a universal feature evaluation method for machine learning models. In the PE2 system, spCas9 activity, RNA folding, and PBS GC content are the top three most important features (Fig. 2a). Interestingly, even though Easy-Prime and the previously published DeepPE [17] are trained with different algorithms and different feature combinations, both programs demonstrate the importance of spCas9 activity and PBS GC content. In the PE3 system, the PAM disruption feature (is_dPAM) and the target mutation position (Target_pos) are two important features following RNA folding and spCas9 activity (Fig. 2b). This is consistent with the previous findings that disrupting the PAM sequence with introduced mutations can improve PE efficiency.

In contrast, the numbers of substitutions, deletions, and insertions are lower-ranked features in both models, which suggests that mutation type does not affect PE efficiency significantly, confirming prime editing to be a versatile tool for different kinds of genome editing [8, 22, 23].

### RNA-folding features associated with PEs

The relationship between RNA folding and PE efficiency has not been fully explored. In our models, the combination of RNA folding related features are the most important and the second most important feature for the PE3 and PE2 systems, which indicates that the pegRNA secondary structure is an important factor for PE. It has been reported that the C-base at the first position in the RTT can pair with G81 in the RNA scaffold [8], which affects the proper gRNA structure required for the interaction between Cas9 and gRNA [24] and leads to lower PE efficiency. Our model showed that the nucleotides at multiple positions in the RTT are important in predicting PE efficiency. To investigate this association further, we formulated the RNA-folding features as the RNA-folding disruption score, defined as the maximal pairing probability between a position in the RTT and the whole scaffold sequence (Fig. 2c). A higher score indicates a stronger interaction between the nucleotide and the RNA scaffold, which can potentially disrupt the RNA secondary structure. We calculated the correlation between the RNA-folding disruption score and the observed PE efficiency based on the data from original prime editing paper [8] for each of the first 16 positions in the RTT (Fig. 2d). The disruption scores for the first five positions showed significant reverse correlation with PE efficiency, indicating that those positions are important for overall PE efficiency. This correlation declines from the sixth to the tenth position and is no longer significant beyond the eleventh position, suggesting that the probability of interaction with scaffold sequences decreases as the distance increases.

### Optimized PE design by Easy-Prime

To demonstrate the effectiveness of Easy-Prime, we used it to design PEs that can install mutations associated with different traits and diseases [25]. We were able to design 136,365 sets of pegRNA and ngRNA to install 89.5% of the 152,351 published GWAS hits (Additional file 1: Fig. S3a). Of those sets, 32,941 are PAM-disrupting edits and 26,873 are predicted to be highly efficient edits (with predicted efficiency of ≥ 40%). For each mutation, an average of 924 sets of pegRNA and ngRNA are searched and optimized (Additional file 1: Fig. S3b).

**Fig. 3:**
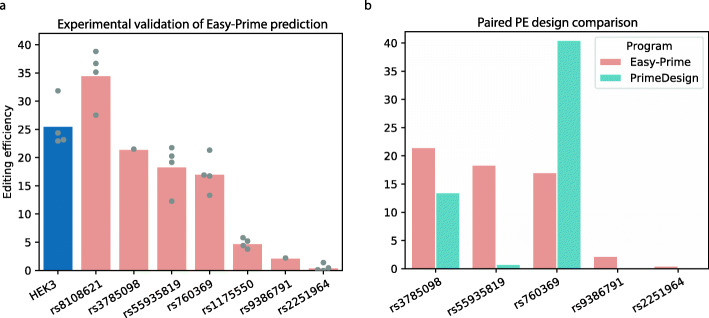
Experimental validation of PE designs by Easy-Prime. **a** Barplot showing the observed editing efficiencies to install a positive control (HEK3 + 1TtoG, blue bar) and 7 blood variants predicted by Easy-Prime (pink bars). Replicates are represented by grey dots. **b** Barplot showing paired PE design comparison between Easy-Prime prediction (pink bars) and PrimeDesign recommendation (cyan bars)

To validate the performance of Easy-Prime, we tested the editing efficiency for installation of 7 variants associated with blood traits [26]. Among the 7 targeted loci, 4 had higher than 10% editing efficiency (Fig. 3a). To further compare the performance between the machine learning based Easy-Prime and the heuristic rules-based programs, we edited 5 loci using pegRNA and ngRNA recommended by PrimeDesign [12]. The sgRNA sequences are exactly the same between Easy-Prime and PrimeDesign. However, the RTT, PBS, and ngRNA sequences selection for the same sgRNA are different (Additional file 3: Table S2). PE designs from Easy-Prime show higher editing efficiency in three loci: rs3785098, rs55935819, and rs9386791 (Fig. 3b). In contrast, PrimeDesign sets show higher efficiency only in rs760369 locus. The editing efficiency is similar between the two programs in rs2251964 locus.

### Interactive web interface of Easy-Prime

**Fig. 4:**
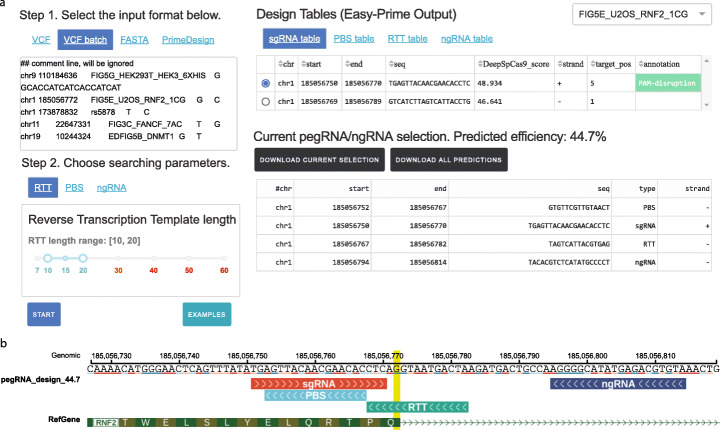
The web portal interface for Easy-Prime. **a** Screenshot of the Easy-Prime web portal (based on DASH [33]). Easy-Prime takes a file in vcf or fasta format as input. It searches and optimizes all individual sgRNA-PBS-RTT-ngRNA combinations and visualizes the gRNAs with the highest predicted efficiency for each input variant. **b** An interactive PE design visualization based on the ProteinPaint genome browser

To enable our model to be easily incorporated into PE design, we developed the Easy-Prime both as a command line program and as a web server (http://easy-prime.cc/) (Fig. 4a). Easy-Prime has four steps (Additional file 1: Fig S4): (1) in the initiation step, it takes a VCF file or a fasta file as input and searches for all possible sgRNAs in a ± 200-bp window; (2) in the expansion step, it enumerates all possible combinations of sgRNA, RTT, PBS, and ngRNA (parameter specifications are provided in Additional file 4: Table S3); (3) in the ranking step, it predicts the editing efficiency using the trained model; and (4) in the visualization step, users can inspect the predicted sequences in an interactive genome browser powered by ProteinPaint [27] (Fig. 4b). The Easy-Prime program provides users with top pegRNA and ngRNA combinations, together with their predicted efficiency. Users can also select and visualize multiple pegRNA/ngRNA combinations.

## Discussion

We developed Easy-Prime, a knowledge-based, one-step solution, to search and optimize the design of PEs automatically. Compared with other approaches, Easy-Prime weighs and combines the contributions of different features to predict editing efficiency and prioritize candidate sequences. In addition to features known to be associated with PE efficiency, Easy-Prime enables us to explore hidden features such as RNA folding and, thus, provides new insights into the mechanism of prime editing.

We have incorporated most of the publicly accessible PE datasets for our model training. However, the current study still has a few limitations. First, the coverage for certain features is sparse and uneven. For example, it is known that the C-base at the first position in the RTT dramatically decreases the editing efficiency of PEs. This is because the C-base can pair with G81 in the RNA scaffold [8] and disrupt the interaction between G81 and Y1356 in Cas9 [24]. This pairing between the RTT and scaffold could destabilize the RNA secondary structure and decrease the activity of PEs [8]. Therefore, 90% (430/477) of the endogenous PE datasets [8] have been designed to avoid using the C-base in the first position. The imbalanced nucleotide composition could explain why the correlation of the first position in the RTT is less than those of the fourth and fifth positions in our model. Also, of the 278 PE3 samples, only seven PE edits are PE3b edits, which explains why the PE3b feature is the least important feature. Second, we used only PE data obtained in HEK293T cells in our PE3 model training. This was because most of the currently available PE data (93%, 1426/1538) were generated in HEK293T cells [8]. Therefore, the cell type–specific features of PE, such as chromatin openness and epigenetic modification, were not investigated in the current study. Another important feature not considered in this study is unwanted editing, including off-target effects and by-product deletions introduced by pegRNA nick sites in the PE3 system [9, 12]. Optimization of PE design to minimize unwanted editing will be a major effort in future development. Because our framework is highly modularized, we expect that both issues can be addressed when more PE data become available in the near future.

## Material and methods

### Public PE data collection

The first PE data source was collected from the DeepPE paper [17], including 46,614 samples that are generated by high-throughput integration system. This PE data was divided into training and testing sets by the authors and we used the same data splits when building and evaluating the PE2 model.

The second PE data source was downloaded from NCBI SRA (BioProject ID: PRJNA565979 [8]). Only HEK293T cell line and PE2-, PE3-, and PE3b-labeled files were used, corresponding to 1426 PE samples including replicates. As the coordinates of the desired edits were not provided in the original prime editing paper [8], we set up a bioinformatic pipeline to re-identify the coordinates. In the first step, we mapped the amplicon sequencing data (fastq files) to hg19 and extracted the consensus amplicon sequences. We then remapped the 3′ extension sequences given in the supplementary file in reference [8] to all of the amplicon sequences mapped in the first step and identified the coordinates of the desired edits. Manual inspection was involved to distinguish SNPs and the target mutation. To quantify the editing efficiency, we used CrisprEsso2 [28] with the same parameters mentioned in reference [8]. Specifically, for single-nucleotide variants, we used the default parameters. For indels, we used the HDR mode. In both cases, we enabled the --discard_indel_reads option. We then merged the replicates with average editing efficiency and split the data into PE2 (*n* = 199) and PE3 (*n* = 278) data. The quantified PE efficiencies were compared to those in the original publication [8], with replication of the main figures. The PE efficiency table and replicated figures are provided in https://github.com/YichaoOU/easy_prime.

The third PE data used for an independent test for Easy-Prime was collected from Hsu et al. [12].

### Feature extraction

Cas9 activity feature is represented as the DeepSpCas9 score [19]. DeepSpCas9 is only available as a web server. We developed a simple python function to simulate web browser events (part of Easy-Prime code).

Oligo features of the PBS and RTT are the sequence length and sequence GC content, which were directly calculated in Python. The PAM-disruption feature is a binary value where 1 means the target mutation disrupts the PAM sequence and 0 means otherwise. PE3b is a binary value where 1 means the spacer of the ngRNA matches to the newly edited sequence and 0 means otherwise. Target mutation features for the numbers of mismatches, insertions, and deletions were computed using skibio (http://scikit-bio.org).

Position features for target mutation (Target_pos) and ngRNA cutting site (ngRNA_pos) are relative positions centered at the pegRNA cutting site, a coordinate system adopted from reference [8]. The target downstream homology (Target_end_flank) represents the number of nucleotides after the target to the RTT end position [12]. RNAplfold [29] was used to compute the RNA secondary structure of the pegRNA. It is composed of the sgRNA, the scaffold, the RTT, and the PBS. Given the base pairing probability *P*(*i*, *j*), where *i* is the *i*th position in the RTT and *j* is the *j*th position in the RNA scaffold, we defined the RNA-folding disruption score *D*(*i*) as follows: *D*(*i*) = max(*P*(*i*, *j*)), ∀ *j* in the RNA scaffold. When the RTT length is less than *i*, position *i* refers to the *i*th position in the 3′ extension sequence (i.e., RTT and PBS).

### Machine learning model

The regression model was implemented using XGBoost [20]. We built PE2 and PE3 models separately (see Fig. 1b). Nested cross-validation was implemented using sklearn [30]. For PE2 model, data was split into training and testing sets based on the train-test-splits from DeepPE for reproducing comparable results. For PE3 model with limited samples, all data from the original prime editing paper [8] was fit into the nested CV framework and the data from Hsu et al. [12] was used for third-party data testing. The outer loop was a 5-fold cross-validation in which the data set was split based on target mutations,defined as the combination of genomic position and target allele. The inner loop was used to tune parameters. XGBoost [20] was tuned for the following parameters: ‘max_depth’: [2, 5, 9, 14], ‘learning_rate’: [0.01,0.1], ‘min_child_weight’: [1, 5, 10], ‘colsample_bylevel’: [0.2,0.6,1], ‘colsample_bytree’: [0.2,0.6,1], ‘subsample’: [0.2,0.6,1], ‘reg_alpha’: [0,0.1,1], and ‘reg_lambda’: [0,1,2]. Feature importance was calculated as the mean absolute of the SHAP value [21].

### Application to GWAS variants

GWAS data were accessed on 5-3-2020 and comprised 185,725 disease/trait associations [25]. Associations that do not have SNP ID were removed. We mapped the SNP ID to dbSNP 152 in hg19. If multiple alternative alleles existed, we expanded the variant to multiple rows. In total, 152,351 GWAS variants were input to Easy-Prime.

### Editing efficiency of designed pegRNA and ngRNA sets

Easy-Prime was used to predict 7 sets of the pegRNA and ngRNA sequences targeting 7 SNV associated with blood traits [26]. These sites were selected randomly after removing common SNPs in HEK293T cells. To directly compare Easy-Prime and PrimeDesign, we generated 5 paired PE designs; one from Easy-Prime prediction and the other one from PrimeDesign recommendation. Raw pegRNA and ngRNA sequences can be found in Additional file 3: Table S2. HEK3 (+ 1 T to G) [8] was used as positive control. HEK293T cells were seeded in a 48-well plate at density of ~ 5 × 10^4^ per well, transfected 24 h post-seeding with PE plasmid, pegRNA plasmid, and nicking gRNA plasmid (750:250:83) and 0.78 μl TransIT (Mirus) (per well). Cells were collected 72 h after transfection and lysed in 150 μl prepared DNA lysis buffer (10 mM Tris-HCl, pH 7.5; 0.05% SDS; 25 μg/ml proteinase K). DNA lysates were incubated at 37 °C for 1 h, followed by an 80 °C enzyme inactivation step for 30 min. For each sample, the target regions were amplified by first round of PCR (PCR1) using gene-specific primers flanking the target sequence. The PCR1 was performed in a 25 μl volume including 100 ng genomic DNA, 1.25 μl of 10 μM each primer, 12.5 μl 2 × Phusion High-Fidelity PCR mix (Thermo) and 0.75 μl DMSO. PCR1 reactions were carried out as follows: 98 °C for 2 min, then 34 cycles of (98 °C for 10 s, 65 °C for 30 s, 72 °C for 30 s), and a final 72 °C extension for 5 min. PCR1 products were purified by electrophoresis in a 1.5% agarose gel or using AMPure beads. In a secondary ‘barcoding’ PCR (PCR2), the amplicons were indexed with primer pairs containing appropriate Illumina forward and reverse adaptor sequences. The 25 μl PCR2 was performed with 10 ng purified PCR1 products, 2.5 μl of 10 μM forward and reverse barcoding primers, 12.5 μl 2 × Phusion High-Fidelity PCR mix (Thermo) and 0.75 μl DMSO. The PCR2 reactions were carried out as follows: 98 °C for 2 min, then 10 cycles of (98 °C for 10 s, 65 °C for 20 s, and 72 °C for 30 s), followed by a final 72 °C extension for 2 min. PCR2 products were purified by electrophoresis with a 1.5% agarose gel and DNA concentration was measured by fluorometric quantification (Qubit, Thermo). Amplicon libraries were sequenced on the Illumina MiSeq instrument according to the manufacturer’s protocols. After demultiplexing, FASTQ files were analyzed using CRISPResso2 with --discard_indel_reads.

## Supplementary Information

**Additional file 1: Fig S1.** The components of a prime editor (PE). **Fig S2.** Nested cross-validation framework. **Fig S3.** Easy-Prime GWAS application. **Fig S4.** Easy-Prime PE design steps.

**Additional file 2: Table S1.** Summary of PE2 and PE3 features. Feature rankings for the PE2 and PE3 model were provided.

**Additional file 3: Table S2.** Paired PE design comparison between Easy-Prime (EP) and PrimeDesign (PD).

**Additional file 4: Table S3.** Easy-Prime parameter specification.

**Additional file 5.** Review history.

## Data Availability

Easy-Prime is a python package freely available under the MIT License in the GitHub repository (https://github.com/YichaoOU/easy_prime [31]) and in a Zenodo repository (10.5281/zenodo.5137926). Easy-Prime web portal is accessible at: http://easy-prime.cc/. All sequencing data have been deposited in the NCBI Gene Expression Omnibus (GEO) with accession number GSE175955 [32]. All data analysis and top PE designs for GWAS variants can be found at https://github.com/YichaoOU/easy_prime.
