# Supplementary material for: Easy-Prime: a machine learning–based prime editor design tool
Source: Genome Biol. 2021 Aug 19;22:235. doi: 10.1186/s13059-021-02458-0 (PMC8377858; doi:10.1186/s13059-021-02458-0)
Supplement: Supplementary file 1 — Additional file 1: Fig S1. The components of a prime editor (PE). Fig S2. Nested cross-validation framework. Fig S3. Easy-Prime GWAS application. Fig S4. Easy-Prime PE design steps. [file 13059_2021_2458_MOESM1_ESM.pdf]

Supplementary files for: Easy-Prime: a machine learning–based prime editor design tool

Yichao Li[1]*, Jingjing Chen[1,2], Shengdar Tsai[1] and Yong Cheng[1,3]*

[1]Department of Hematology, St. Jude Children's Research Hospital, Memphis, Tennessee, USA

[2]Integrated Biomedical Sciences Program, University of Tennessee Health Science Center, Memphis, TN, USA

[3]Department of Computational Biology, St. Jude Children's Research Hospital, Memphis, TN, USA

* To whom correspondence should be addressed. Email: Yichao.Li@stjude.org, Yong.Cheng@stjude.org

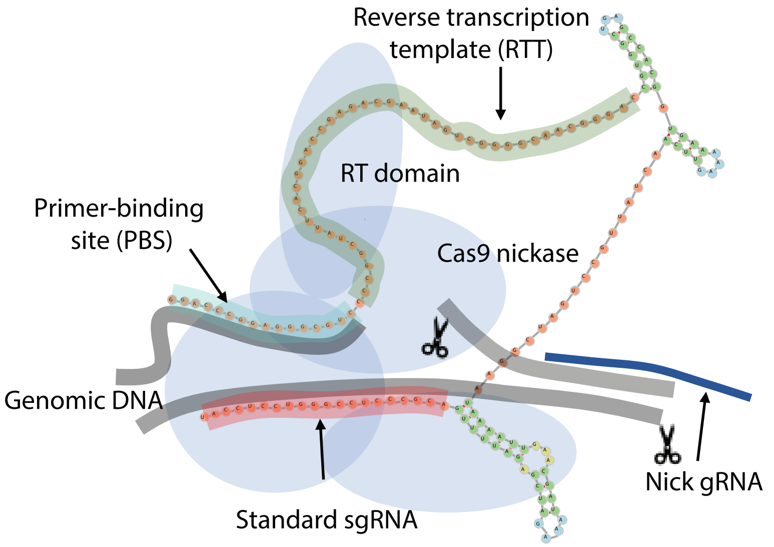

Supplementary Figure 1. The components of a prime editor (PE). PE consists of a Cas9 nickase fused with reverse transcriptase (blue bubble) and a pegRNA. The pegRNA sequence contains a standard sgRNA (red curve), a primer-binding site (PBS, cyan curve) that primes the RT reaction, and a reverse-transcription template (RTT, green curve) that copies the desired edit. Another standard sgRNA, called nick gRNA (blue curve), can nick the non-edited strand to further increase the editing efficiency. Grey lines are DNA at the targeted locus.

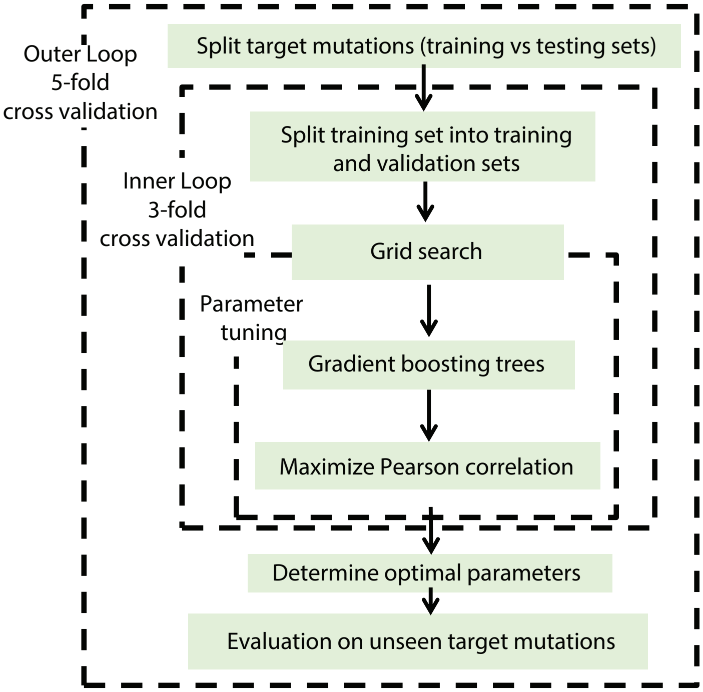

Supplementary Figure 2. Nested cross-validation framework. The outer cross-validation loop randomly split the data into 5 folds, of which 4 folds were used for training and the remaining fold was used for testing. Then, for each training set, the optimal parameters were determined using the inner loop, which was another 3-fold cross-validation consisting of a grid search of parameters. The final reported Pearson correlation was the mean Pearson correlation from the outer 5-fold cross-validation. For reporting, both Spearman and Pearson correlation were calculated.

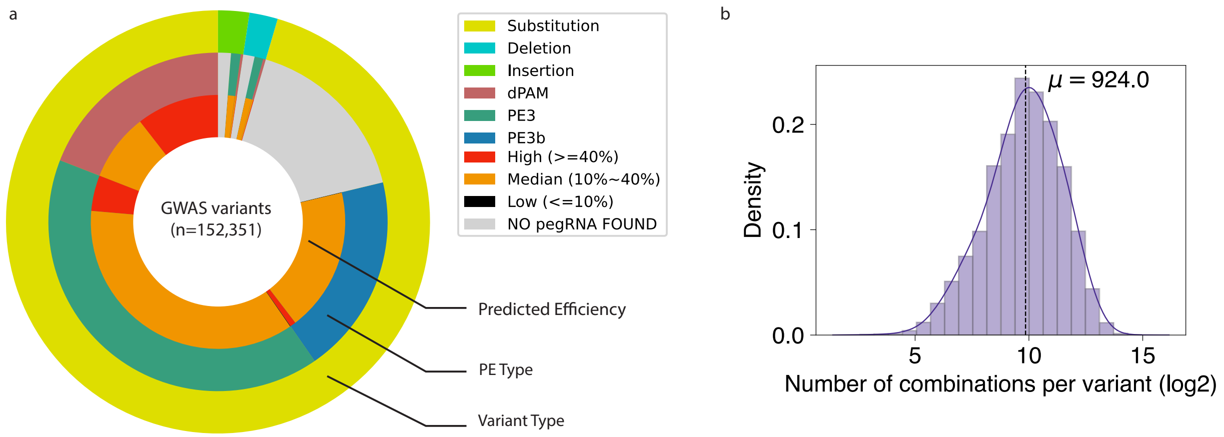

Supplementary Figure S3. Easy-Prime GWAS application. a, A nested pie-chart showing the prime editors (PEs) for GWAS variants designed by Easy-Prime. The outer layer represents the different variant types: substitution, deletion, and insertion. The middle layer represents the PE types: PAM disruption (dPAM), PE3, and PE3b. The inner layer represents the predicted efficiency: high (≥40%), median (between 10% and 40%), and low (≤10%). **b**, Histogram showing the distribution of the numbers of optimized sets of pegRNA and ngRNA per variant. Average number is 924.

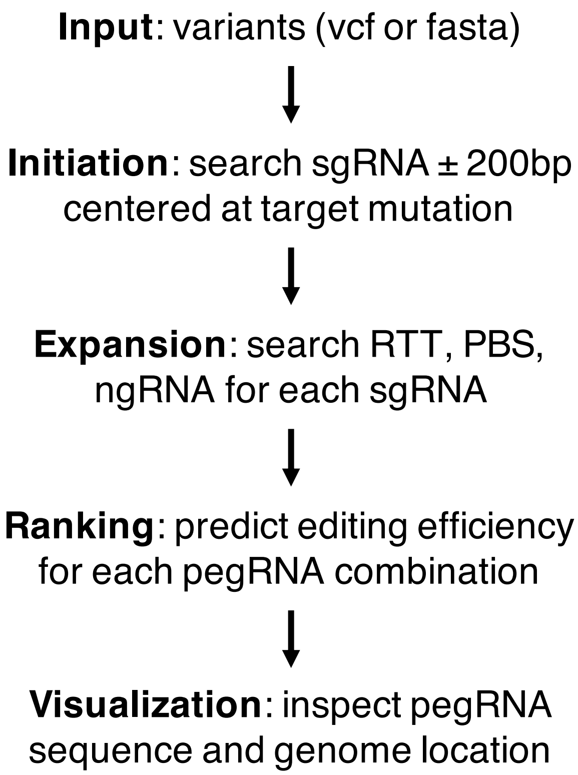

Supplementary Figure S4. Easy-Prime PE design steps.
