# Supplementary material for: Easy-Prime: a machine learning–based prime editor design tool
Source: Genome Biol. 2021 Aug 19;22:235. doi: 10.1186/s13059-021-02458-0 (PMC8377858; doi:10.1186/s13059-021-02458-0)
Supplement: Supplementary file 4 — Additional file 4: Table S3. Easy-Prime parameter specification. [file 13059_2021_2458_MOESM4_ESM.docx]

**Table S3. Easy-Prime parameter specification.**

| Parameter | Default values | References |
| --- | --- | --- |
| PBS_length | [10,15] | 13nt^1^, 12-14nt^2^ |
| RTT_length | [10,20], up to 50nt if not found, step size = 5 | 10-16nt^1^, 10-20nt^2^ |
| ngRNA_pos (nicking distance) | [0-100], up to 150nt if not found, step size = 20 | 50nt^1^, 0-100nt^2^ |
| Target_end_flank (downstream homology) | Minimum 5 nt | Minimum 5 nt^2^ |
| Target_pos | [0-10], up to 30 if not found, step size = 5 | 98% data fall in this range (n=477)^1^ |
| PAM disruption | Choose a pegRNA that disrupts the PAM sequence when possible | Recommend PAM disruption whenever possible^1^ |

1. Anzalone, A. V. *et al.* Search-and-replace genome editing without double-strand breaks or donor DNA. *Nature* **576**, 149–157 (2019).

2. Hsu, J. Y. *et al.* PrimeDesign software for rapid and simplified design of prime editing guide RNAs. *Nat. Commun.* **12**, 1034 (2021).
