# Supplementary material for: Easy-Prime: a machine learning–based prime editor design tool
Source: Genome Biol. 2021 Aug 19;22:235. doi: 10.1186/s13059-021-02458-0 (PMC8377858; doi:10.1186/s13059-021-02458-0)
Supplement: Supplementary file 5 — Additional file 5. Review history. [file 13059_2021_2458_MOESM5_ESM.docx]

Review History

**First round of review**

**Reviewer 1**

**Were you able to assess all statistics in the manuscript, including the appropriateness of statistical tests used?** Yes, and I have assessed the statistics in my report.

**Were you able to directly test the methods?** Yes.

**Comments to author:**

Li et al developed a machine learning model to accurately predict PE activities. The model was further used to develop "Easy-Prime", a user-friendly program for PE design. Generally, the manuscript is well written, and the relevant literatures are cited (e.g. two tools pegFinder and PrimeDesign that are similar to this study). The Easy-Prime program predicts and ranks potential pegRNA and ngRNA, so that users can choose the top pegRNA and ngRNA combinations. However, I don't think this study is well enough because some of the results are not convincing.

Marjor Comments:
1) As is shown in Fig. 1d, the model performs well for PE3. However, the prediction on PE2 efficacy seems poor. Do you have any idea on such difference? Considering that the PE2 system is quite different from the PE3 system, I don't think it proper to train a combined model for them.

2) As for the features. PE2.vs.PE3 feature was used to indicate whether or not the PE design contains ngRNA. Why not just develop two models for them? Moreover, because PEs are developed from CRISPR systms, the CRISPR editing efficacy is effective for PE editing efficacy prediction (refer to DeepPE). I suggest a further development.

3) The author train the model with 1426 published amplicon sequencing datasets. It is far from satisfactory. The newly published data can be used (refer to DeepPE).

4) The model was evaluated by cross-validation. However, a valid model requires an independent testing dataset.

Minor Comments:
1) Because PE is a recently-developed genome editing tool, a more clear introduction may be required for a broad audience.

2) The prediction accuracy was shown as "R = 0.78". However, the authors did not specifify what correlation is used (Pearson or Spearman?). Please show the spearman correlation.

**Reviewer 2**

**Were you able to assess all statistics in the manuscript, including the appropriateness of statistical tests used?** There are no statistics in the manuscript.

**Were you able to directly test the methods?** No.

**Comments to author:**

The authors use data from Anzalone et al 2019 and learn a computational model to predict prime editor (PE) designs with good efficacy and experimentally test several designs. The major concern is whether this model is better than the qualitative recommendations implemented in PrimeDesign by Hsu et al 2021.

Major comments

1. A direct comparison between this model and PrimeDesign would help readers to understand the significance of this model. Can the authors determine and describe how recommendations differ between their machine learning model and the heuristic rules based on the original PE publication and implemented as PrimeDesign?

Authors should clearly describe which parameters are new (e.g., RNA folding disruption) and how each of the significant novel features contributed to the improvement of this model vs. the heuristic rules implemented in PrimeDesign. Conversely, it would also be informative if the authors could list any features excluded from this model that were implemented in PrimeDesign.

2. Another key question is whether the recommended designs are improved with this model compared to the recommendations as implemented in PrimeDesign. The authors could survey recommendations from Prime Design to find how often the top ranked design differs from Easy Prime. For recommendations that do differ, a small number should be experimentally tested to determine which design is superior.

3. Suggest the authors include a table with all feature information, RTT sequence, PBS, pegRNA,  ngRNA, along the true efficacy and the predicted efficacy.


4. In Figure 1D,  there is lack of evidence that the predicted efficacies are correlated with the observed ones for the PE2 system. We know PE2 designs are not as good, so why include them in the training dataset unless including them improves the prediction for PE3. Can the authors demonstrate the benefit of including PE2 system in the model?

5.  In Supplementary S4d, there is small number of data points and the line seems to be mostly influenced by a few points at the lower left corner. In addition, the efficacy for all points is moderate to low, which indicates that PEs in the high efficacy range are not being tested.

6. The authors don't mention impact of dual nicks on formation of undesired deletions. This should be included in the discussion.

7. The panel "PE design visualization for:" at http://easy-prime-test-dev.us-west-2.elasticbeanstalk.com is blur with a test run. Not sure whether this is a bug.

8. The command tool does not run after running the following commands.

git clone https://github.com/YichaoOU/easy_prime

cd easy_prime/test

easy_prime -h

-bash: easy_prime: command not found

9. Installation failed with conda create

conda create -n genome_editing -c cheng_lab easy_prime

PackagesNotFoundError: The following packages are not available from current channels:

 - python_abi=3.7[build=*_cp37m]

Current channels:

 - https://conda.anaconda.org/cheng_lab/osx-64
 - https://conda.anaconda.org/cheng_lab/noarch
 - https://repo.anaconda.com/pkgs/main/osx-64
 - https://repo.anaconda.com/pkgs/main/noarch
 - https://repo.anaconda.com/pkgs/r/osx-64
 - https://repo.anaconda.com/pkgs/r/noarch

To search for alternate channels that may provide the conda package you're
looking for, navigate to

   https://anaconda.org

and use the search bar at the top of the page.


Minor comments

1. Does the Table output allow for multiple selections for each target so user can consider and/or test more than one?

2. Hsu et al reference for PrimeDesign should be updated to Nat Comm publication.

3. Line 94 : should reword to clarify that the "previous report" was the original description of prime editor and dataset.

4. Please indicate how the specific GWAS SNPs were selected for analysis in Fig S4.

**Authors Response**

**Point-by-point responses to the reviewers’ comments:**

Reviewer 1

1) As is shown in Fig. 1d, the model performs well for PE3. However, the prediction on PE2 efficacy seems poor. Do you have any idea on such difference? Considering that the PE2 system is quite different from the PE3 system, I don't think it proper to train a combined model for them.

**Response: We agree with the reviewer that the performance of the PE2 system in our previous manuscript was not as good as that of the PE3 system. This is mainly due to the limited PE2 data available during that time. Following the reviewer's suggestion, we have 1) included 46,614 PE2 data from Kim et al., 2021, the DeepPE paper¹ and 2) retrained Easy-Prime with different models for PE2 and PE3 systems.  We have updated the text (line 89-103) and Fig. 1 and Fig. 2 based on the new models.**

2) As for the features. PE2.vs.PE3 feature was used to indicate whether or not the PE design contains ngRNA. Why not just develop two models for them? Moreover, because PEs are developed from CRISPR systms, the CRISPR editing efficacy is effective for PE editing efficacy prediction (refer to DeepPE). I suggest a further development.

**Response: We thank the reviewer for the thoughtful suggestions. We have developed and trained two separate models for PE2 and PE3 in the revised manuscript. We also included the standard spCas9 editing efficacy (represented by DeepSpCas9 score) in the new model as suggested by the reviewers. We did observe improved performance on both systems, especially for PE2. Consistent with the reviewer’s suggestion, the Cas9 editing efficacy ranks 1st and 2nd among the most important features in PE2 and PE3 systems (Fig. 2), respectively.**

3) The author train the model with 1426 published amplicon sequencing datasets. It is far from satisfactory. The newly published data can be used (refer to DeepPE).

**Response:  We thank the reviewer for this helpful suggestion. We have included 46,614 PE2 data from DeepPE in our new training.**

4) The model was evaluated by cross-validation. However, a valid model requires an independent testing dataset.

**Response:  We agree with the reviewer. In the revised manuscript, we added 33 PE3 data generated recently by Hsu et al.,² as an independent testing dataset to evaluate the performance of our model. The correlation between predicted and observed editing efficiency is 0.5 (Spearman’s correlation). We have included this result in line 100-103 and new Fig. 1d.**

Minor Comments:

1) Because PE is a recently-developed genome editing tool, a more clear introduction may be required for a broad audience.

**Response:  We thank the reviewer for this suggestion and added more details on Prime Editing in the introduction (line 48-56).**

2) The prediction accuracy was shown as "R = 0.78". However, the authors did not specifify what correlation is used (Pearson or Spearman?). Please show the spearman correlation.

**Response: We apologize for the lack of clarity. The correlation shown in the previous version was Pearson’s correlation. Following the reviewer’s suggestion, we included both Spearman (shown as “R”) and Pearson correlation (shown as “r”) in the revised manuscript (see Figure 1 and line 99-103).**

Please consider the following questions:

-Is the Software likely to be of broad utility? Is it easy to install and use?

**Response: Due to broad application of Prime editing, we feel Easy-Prime will be widely used. We have designed both command line and web-interface versions of Easy-Prime. The command line version can be easily installed in Linux or Mac systems via Conda (https://easy-prime.readthedocs.io/en/latest/content/Installation.html). The web version can be accessed at http://easy-prime.cc/.**

-Does the software represent a significant advance over previously published tools (as demonstrated by direct comparison with available related software)?

**Response: To our best knowledge, among all the Prime editing design software, Easy-Prime is the only one that can optimize different combinations of pegRNA and ngRNA sequences to improve editing efficiency.**

-Please indicate briefly the novel features and/or advantages of the software and/or please reference the relevant publications and which alternatives, if any, it should be compared with.

**Response: Easy-prime and other PE design tools are all developed roughly around the same time. Besides commonly recommended features, Easy-Prime is the first model to incorporate both RNA folding and cas9 activity in the PE design.**

**Unlike other PE design tools, Easy-Prime provides an automated system to rank pegRNA/ngRNA design and output the best design based on thousands of PE data.**

-Are the methods appropriate to the aims of the study, are they well described, and are necessary controls included?

**Response:  All the methods are properly documented. Positive and negative controls are also included.**

-Are the conclusions adequately supported by the data shown?

**Response: We have used multiple independent approaches to assess the performance of Easy-Prime, including cross-validation, third-party dataset validation and experimental validation.**

-Are sufficient details provided to allow replication and comparison with related analyses that may have been performed?

**Response: We have provided detailed method sections for each step of analysis. We also provided the source code for Easy-Prime as well as raw data in https://github.com/YichaoOU/easy_prime.**

-Is the paper of broad interest to others in the field, or of outstanding interest to a broad audience of biologists?

**Response: Yes. Prime editing has been applied in human, mouse, and plants and show promising results. We expect even more applications in the near future.**

Reviewer 2

The authors use data from Anzalone et al 2019 and learn a computational model to predict prime editor (PE) designs with good efficacy and experimentally test several designs. The major concern is whether this model is better than the qualitative recommendations implemented in PrimeDesign by Hsu et al 2021.

Major comments

1. A direct comparison between this model and PrimeDesign would help readers to understand the significance of this model. Can the authors determine and describe how recommendations differ between their machine learning model and the heuristic rules based on the original PE publication and implemented as PrimeDesign?

Authors should clearly describe which parameters are new (e.g., RNA folding disruption) and how each of the significant novel features contributed to the improvement of this model vs. the heuristic rules implemented in PrimeDesign. Conversely, it would also be informative if the authors could list any features excluded from this model that were implemented in PrimeDesign.

**Response: The reviewer raised an important question. Overall, compared with PrimeDesign, Easy-prime includes more features in its machine learning-based model and also weighs the contribution of each feature based on the training using thousands of published PE data.**

**Specifically, our model includes classic PE design features, i.e. features that have been examined in initial PE paper from Anzalone et. al, such as target position, PBS length, RTT length, ngRNA distance and PAM disruption.  We also include additional important features, such as RNA folding features and spCas9 activity. Interestingly, these two features are the top two most important contributors to PE editing efficiency in our models. In contrast, none of the features used in the PrimeDesign were excluded in Easy-Prime. As suggested by the reviewer, we have updated the text (line 122-125) to emphasize the unique features of Easy-Prime and added Supplementary Table 1 to list all the features in the Easy-prime model, their contribution to the overall performance and whether they are included in other software.**

2. Another key question is whether the recommended designs are improved with this model compared to the recommendations as implemented in PrimeDesign. The authors could survey recommendations from Prime Design to find how often the top ranked design differs from Easy Prime. For recommendations that do differ, a small number should be experimentally tested to determine which design is superior.

**Response:  We thanks the reviewer for this thoughtful suggestion. To compare the recommendation between PrimeDesign and Easy-Prime, we performed prime editing in 5 loci with pegRNA and ngRNA sets designed by the two programs. The sgRNA sequences recommended by the two programs are the same. Most of the difference is due to the design on RTT, PBS, and ngRNA. The designs of Easy-Prime show higher editing efficiency in three targeted loci. PrimeDesign shows higher editing efficiency in one locus. There is also one locus, the design from neither program can introduce detectable editing. We have reported this new results in line 149-159 and Figure 3.**

3. Suggest the authors include a table with all feature information, RTT sequence, PBS, pegRNA,  ngRNA, along the true efficacy and the predicted efficacy.

**Response: We thank the review for this suggestion. We have added a new table (Supplementary Table 2) that provides all the information.**

4. In Figure 1D,  there is lack of evidence that the predicted efficacies are correlated with the observed ones for the PE2 system. We know PE2 designs are not as good, so why include them in the training dataset unless including them improves the prediction for PE3. Can the authors demonstrate the benefit of including PE2 system in the model?

**Response: The reviewer raised an important question. In fact, review 1 also has the same concern. We believe the lack of correlation in PE2 is due to the limited number of training samples. Thus, in the revised manuscript, we have included 46,614  PE2 data from Kim et al., 2021, DeepPE paper¹and re-trained the two systems separately. The new PE2 model shows improved performance. We have updated the manuscript (line 89-103) in the result section and in Fig. 1 and Fig. 2.**

5.  In Supplementary S4d, there is small number of data points and the line seems to be mostly influenced by a few points at the lower left corner. In addition, the efficacy for all points is moderate to low, which indicates that PEs in the high efficacy range are not being tested.

**Response:  We agree with the reviewer that the correlation will be more stable with larger experimental validation datasets. Another potential explanation is that Prime Editing is still a relative new technology, how to optimize the experiment remain challenging for most labs. Thus, the true editing potential may be underestimated. For example, only 6 of the 20 validated targeted in PrimeDesign paper show higher than 10% editing efficiency and the highest efficiency is only about 35%. In the revised version, we have removed the correlation analysis due to the potential lack of stability. Instead, we used the 33 PE data (correspond to the 20 validated sites, some sites have multiple pegRNA and ngRNA combinations) from PrimeDesign as a third-party independent validation. The correlation between predicted and observed editing efficiency is 0.5 (Spearman’s correlation) (see Line 100-103 and Figure. 1d).**

6. The authors don't mention impact of dual nicks on formation of undesired deletions. This should be included in the discussion.

**Response: We thank the reviewer for this thoughtful comment. As the reviewer pointed out, we did observe low frequency deletions near pegRNA nick sites in both published datasets3 and our own data. However, quantifying and modeling by-product PE editing such as deletions and off-target editing are not accessed in our mode mainly due to relative sparse available datasets. We have discussed this caveat in the discussion (line 194-197)**

7. The panel "PE design visualization for:" at  http://easy-prime-test-dev.us-west-2.elasticbeanstalk.com/ is blur with a test run. Not sure whether this is a bug.

**Response: We apologized for this potential bug. We have updated the web interface (http://easy-prime.cc/ ). In addition, we also include a “check running status” button in the new web interface to output error messages.**

8. The command tool does not run after running the following commands.

git clone  https://github.com/YichaoOU/easy_prime

cd easy_prime/test

easy_prime -h

-bash: easy_prime: command not found

9. Installation failed with conda create

conda create -n genome_editing -c cheng_lab easy_prime

PackagesNotFoundError: The following packages are not available from current channels:

  - python_abi=3.7[build=*_cp37m]

Current channels:

To search for alternate channels that may provide the conda package you're

looking for, navigate to

and use the search bar at the top of the page.

**Response: We apologize for this error. It was likely caused by the Conda version and/or operating system. Easy-Prime requires conda version >= 4.9 in Linux and Mac. Easy-Prime is not available in Windows OS because ViennaRNA, the package Easy-prime uses to calculate the RNA folding, does not support windows OS.  We have added a statement on this in both the GitHub README page and the Easy-Prime installation tutorial webpage. To avoid other potential confusion during installation, we have provided step-by-step installation tutorials here: https://easy-prime.readthedocs.io/en/latest/content/Installation.html**

Minor comments

1. Does the Table output allow for multiple selections for each target so user can consider and/or test more than one?

**Response: Thanks for the suggestion. We have now implemented this feature and users can select any number of combinations to download as well as visualization.**

2. Hsu et al reference for PrimeDesign should be updated to Nat Comm publication.

**Response: Updated.**

3. Line 94 : should reword to clarify that the "previous report" was the original description of prime editor and dataset.

**Response: We apologize for the lack of clarity. We have replaced all “previous report” and similar words with “the original study of prime editor”.**

4. Please indicate how the specific GWAS SNPs were selected for analysis in Fig S4.

**Response: Since GWAS SNPs are common variants, we first removed variants that already exist in HEK293T cells. We then randomly selected targeted loci for variant installation.  Some targeted loci are excluded due the technique issues, such as difficulties in pegRNA expression cassette constructions and/or PCR amplification. We have updated the text in line 272-276.**

Reference

1.	Kim, H. K. et al. Predicting the efficiency of prime editing guide RNAs in human cells. Nat. Biotechnol. 39, 198–206 (2021).

2.	Hsu, J. Y. et al. PrimeDesign software for rapid and simplified design of prime editing guide RNAs. Nat. Commun. 12, 1034 (2021).

3.	Anzalone, A. V. et al. Search-and-replace genome editing without double-strand breaks or donor DNA. Nature 576, 149–157 (2019).

**Second round of review**

**Reviewer 1**

This is a nice article after well revision following reviewers' comments. The authors developed machine learning models to predict PE editing efficiency and design optimized pegRNA. Prime editing is developing rapidly, and several PE design tools have been developed to facilicate pegRNA design and prime editing experiments. The authors used novel features and limited data to develop machine learning models, which achieved considerable performance. The optimized PE was experimentally validated, which showed high editing efficiency. This is a good work, and I recommend its publication after minor revision.

1. Lines 112-114: "In PE3 system, the PAM disruption feature (is_dPAM) and the target mutation position (Target_pos) are two important features following RNA folding and spCas9 activity (Fig. 2b)." Figure 2b indicated that ngRNA_pos is more important than Target_pos. What is ngRNA_pos? Is it similiar to Target_pos?

2. Lines 157-158: "In contrast, PrimeDesign sets show higher efficiency only in rs760369 locus." I am curious about Easy-Prime score of the PrimeDesign rs760369 PE. How does it rank? Is it among the top ranking PEs designed by Easy-Prime?

3. The Easy-Prime website shows just the top pegRNA and its efficiency. I suggest the authors to show the top ranking pegRNAs (i.e. the top N pegRNAs) and their scores, so that users can make their choice.

4. The authors collected PE data obtained in the HEK293T cell line for training and testing, while excluded data from other cell lines. PE data are limited, so the authors may make good use of those from cell lines other than HEK293T. Can these data be used as an independent testing dataset to indicate the influence of cell type and epigenetic features?

**Reviewer 2**

Authors did address most of previous comments.

1. Now that the authors have included the larger DeepPE dataset, the performance for PE2 should be more reliable. However, it’s not clear whether the performance of EasyPE for PE2 is better than DeepPE, which uses the same algorithm with similar dataset and features. According to the DeepPE publication, Spearman’s correlations were between 0.47 and 0.81 using independent test dataset with DeepPE’s computational models for predicting efficiencies of pegRNAs with different lengths of primer binding sites and reverse transcriptase templates for edits of various types and positions. 

2. Authors do not include DeepPE in their discussion of other tools for PE design (i.e. line 58) except to note the paper as a datasource. Additional tools in the literature should also be mentioned such as PE-designer.

3. The authors did generate a predictive model for PE3, which is not available for DeepPE. Unfortunately, once PE3 dataset is separated, the training/test dataset becomes even smaller. It is not clear whether this small dataset is sufficient to make a reliable predictive model similar to that for PE2. The limited data in Figure 3 does not make a compelling case for superior designs.

**Authors Response**

**Point-by-point responses to the reviewers’ comments:**

Reviewer 1

This is a nice article after well revision following reviewers' comments. The authors developed machine learning models to predict PE editing efficiency and design optimized pegRNA. Prime editing is developing rapidly, and several PE design tools have been developed to facilicate pegRNA design and prime editing experiments. The authors used novel features and limited data to develop machine learning models, which achieved considerable performance. The optimized PE was experimentally validated, which showed high editing efficiency. This is a good work, and I recommend its publication after minor revision.

**We thank the reviewer for the positive comments.**

1. Lines 112-114: "In PE3 system, the PAM disruption feature (is_dPAM) and the target mutation position (Target_pos) are two important features following RNA folding and spCas9 activity (Fig. 2b)." Figure 2b indicated that ngRNA_pos is more important than Target_pos. What is ngRNA_pos? Is it similiar to Target_pos?
**Response: The ngRNA_pos is the distance between the nick sites of ngRNA and sgRNA. Positive or negative ngRNA_pos indicates that the ngRNA nick site is downstream or upstream of sgRNA nick site, respectively. This coordinate system was adopted from the original prime editing paper and illustrated in Figure 1a. We initially described these terms in the Introduction section. To avoid confusions, we added the descriptions of Target_end_flank, Target_pos, ngRNA_pos in the legend of Figure 2 as follows:

“Target_end_flank: number of nucleotides from target mutation to the end of RTT sequence. Target_pos: distance between target mutation and sgRNA nick site. ngRNA_pos: distance between ngRNA nick site and sgRNA nick site”.**

2. Lines 157-158: "In contrast, PrimeDesign sets show higher efficiency only in rs760369 locus." I am curious about Easy-Prime score of the PrimeDesign rs760369 PE. How does it rank? Is it among the top ranking PEs designed by Easy-Prime?
**Response: The Easy-Prime ranks PrimeDesign rs760369 PE as top 28%, with a predicted editing efficiency of 31.2%, which is a bout 10% less than the predicted maximal efficiency (42.2%)**

3. The Easy-Prime website shows just the top pegRNA and its efficiency. I suggest the authors to show the top ranking pegRNAs (i.e. the top N pegRNAs) and their scores, so that users can make their choice.
**Response: We thank the reviewer for this suggestion. We now add an option to download the top 5 predicted pegRNA/ngRNA combinations (see Figure 1 below).

Figure 1. Screenshot of Easy-Prime to show the addition of the “top5 prediction” download button.**

4. The authors collected PE data obtained in the HEK293T cell line for training and testing, while excluded data from other cell lines. PE data are limited, so the authors may make good use of those from cell lines other than HEK293T. Can these data be used as an independent testing dataset to indicate the influence of cell type and epigenetic features?
**Response: We thank the review for this thoughtful suggestion. Compared with HEK293T cells, the editing efficiency of PE in other cell lines is significantly lower (see Figure2 below). The authors of the original PE paper mentioned that the PE method was not fully optimized in other cells. Thus, the variation of PE results in different cell types is actually caused by both cell type difference and technique limits. Currently, it is difficult to discriminate between the contributions of two factors.


Figure 2: Barplot showing the editing efficiency in four different cell lines. Data source: Anzalone 2019. Each dot is an individual sample.**


Reviewer 2

1. Now that the authors have included the larger DeepPE dataset, the performance for PE2 should be more reliable. However, it’s not clear whether the performance of EasyPE for PE2 is better than DeepPE, which uses the same algorithm with similar dataset and features. According to the DeepPE publication, Spearman’s correlations were between 0.47 and 0.81 using independent test dataset with DeepPE’s computational models for predicting efficiencies of pegRNAs with different lengths of primer binding sites and reverse transcriptase templates for edits of various types and positions.
**Response: We thank the reviewer for this important comment. Easy-Prime and DeepPE are two complementary programs. They share some common features such as SpCas9 activity and RTT/PBS GC and length in the prediction model. Meanwhile, they also have their unique features. For example, the RNA-folding feature is unique to Easy-Prime. Interestingly, the top features identified by both programs are highly consistent. In addition, as the reviewer mentioned, only Easy-Prime can be used for PE3 prediction, as this function is not supported by DeepPE.**


2. Authors do not include DeepPE in their discussion of other tools for PE design (i.e. line 58) except to note the paper as a datasource. Additional tools in the literature should also be mentioned such as PE-designer.
**Response: We now include the citation of DeepPE and PE-designer.**

3. The authors did generate a predictive model for PE3, which is not available for DeepPE. Unfortunately, once PE3 dataset is separated, the training/test dataset becomes even smaller. It is not clear whether this small dataset is sufficient to make a reliable predictive model similar to that for PE2. The limited data in Figure 3 does not make a compelling case for superior designs.
**Response: Even though we have collected most published PE3 data for Easy-Prime training, we do agree with the reviewer that it is still relatively small compared to the PE2 data generated in the DeepPE study. Therefore, we undertook rigorous evaluation for the Easy-Prime performance. First, the comparable performance of Easy-Prime and DeepPE on PE2 data validated the robustness of the Easy-Prime algorithm and feature representation. Next, the Easy-Prime PE3 model was evaluated using Nested Cross-validation with data from Anzalone 2019. Finally, an independent data set from Hsu 2021 was used a third-party validation, which shows similar performance as the hold-out validation of DeepPE on PE2 dataset (i.e., type-test). (0.5 vs 0.47). Taken together, our PE3 prediction model shows robust performance in all the validation. As the reviewer mentioned, the most convincing evidence will be a larger PE3 data set generated by a high-throughput approach, which is currently still not available. We discussed this limitation in the manuscript. Like all machine learning based approaches, when more PE3 data become available in the future, we can re-train our model to further improve its performance.**
